# One-Step Air Spraying of Structural Coating on Cu Alloy as Superhydrophobic Surface for Enhanced Corrosion Resistance and Anti-Icing Performance

**DOI:** 10.3390/ma17184485

**Published:** 2024-09-12

**Authors:** Ben Li, Xuewu Li

**Affiliations:** 1 Engineering Research Center of Additive Manufacturing Aeronautical Materials of Henan Province, Nanyang Institute of Technology, Nanyang 473004, China; 2 School of Mechanical Engineering, Xi’an University of Science and Technology, Xi’an 710054, China

**Keywords:** Cu alloy, Al_2_O_3_-PDMS composite coating, air spraying, superhydrophobic surface, corrosion resistance, anti-icing performance

## Abstract

With the development of modern technology, the construction industry, and navigation technology, the metal Cu alloy has become an important metal material in mainstream industrial applications. As an indispensable basic metal material in the field of science and technology, its problem with corrosion is still a long-term problem that scientists have been working to solve. In this paper, air spraying technology is used to prepare an Al_2_O_3_-PDMS composite coating. By adjusting the content of Al_2_O_3_, the surface of the Cu alloy can reach different wetting states. The results show that the corrosion potential of the as-prepared superhydrophobic Al_2_O_3_-PDMS coating increases by 70 mV compared with the substrate, the corrosion current density decreases by one order of magnitude, and the impedance modulus increases from 2000 to 12,000 Ω⋅cm^2^, indicating a significantly enhanced corrosion resistance. It also possesses excellent anti-pollution and anti-icing behaviors, thereby allowing them to work in harsh industrial conditions.

## 1. Introduction

Cu alloys are widely used in many fields such as aviation, aerospace, precision manufacturing, machining, and transportation because of its excellent electrical, thermal, and mechanical properties [1]. And Cu alloys play a very important role in daily human life and various processing industries [2]. In general, Cu alloys possess relatively stable physical and chemical properties [3]. However, Cu alloys fail in corrosive environments [4], which seriously affects their physical structure and service properties [5]. Surface engineering and protection technology are commonly used to solve the problem with corrosion of Cu alloys [6]. However, the traditional solutions, such as corrosion inhibitors [7], mechanical alloying [8], laser treatment [9], surface oxidation [10], and friction stir welding [11], always have problems with complex operation, high cost, unfavorable environmental conditions, and poor controllability [12]. Therefore, it is very important to seek a simple, efficient, low-cost, controllable, and environmentally friendly corrosion protection method for Cu alloys.

In recent years, inspired by the lotus leaf effect [13], superhydrophobicity has provided an effective way [14] to solve the corrosion problem with Cu alloys because of their excellent liquid displacement [15]. Meanwhile, a superhydrophobic surface with self-cleaning [16], anti-fouling [17], drag-reducing [18], anti-frost [19], and other aspects [20] has an excellent effect, which has great potential in industrial applications [21]. Accordingly, based on its bionic nature, the preparation of a superhydrophobic coating on a copper substrate is of great scientific significance and industrial value for solving corrosion problems [22], which will also further expand the scope of industrial applications of copper-based materials.

The selection of an appropriate method is crucial in the fabrication of a superhydrophobic coating on a copper substrate. At present, the commonly used coating strategies include electrodeposition [23], the sol–gel process [24], electroplating [25], etching [26], and chemical vapor deposition [27]. In addition, the combination of sintering and a spraying strategy has also been reported for preparing organic–inorganic multilayer superhydrophobic coatings on copper surfaces [28]. Dealloying–forming and solution–immersion processes have also been used to adhere strong nickel and PDMS materials together, thus providing superhydrophobic coating protection for copper substrates [29]. Such coating technologies have certain issues, to a greater or lesser extent, in terms of preparation cost, structural stability, and preparation scale. In particular, the preparation process of superhydrophobic coatings by the multi-step method is relatively complex.

In this paper, a simple, low-cost, and large-scale air spraying technique is developed to fabricate a micro–nano-structure coating with layered characteristics on a T2 Cu alloy. The superhydrophobic surface is composed of the micro-structure and low-surface-energy substance [13]. Herein, aluminum ceramic particles are used as spraying solute particles due to their excellent mechanical strength, chemical stability, wear resistance, deformation resistance, easy cleaning, and chemical resistance [30]. Meanwhile, PDMS is a polymer with advantages such as heat resistance, cold resistance, low viscosity variation with temperature, waterproofing, and low surface tension [31]. The combination of aluminum microparticles with low-surface-energy PDMS is expected to achieve an excellent superhydrophobic effect. In addition, the wettability of the spray coating is optimized by adjusting the composition of the coating. The structural coating prepared by air spraying can capture more air phases when it meets liquid, thus producing an “air cushion” effect to hold up the water droplets. Ultimately, the viscous resistance to water nearly disappeared, thus showing excellent superhydrophobic characteristics, anti-pollution behavior, corrosion resistance, and anti-icing performance. This work provides a promising strategy for expanding the application of Cu alloys, especially in the engineering field.

## 2. Sample Preparation and Characterization

### 2.1. Materials

The commercially available T2 Cu alloy plates (20 mm × 20 mm × 5 mm) were used, and the corresponding chemical compositions are listed in Table 1. Other reagents of analytical grades were obtained from Sinopharm Chemical Reagent Co., Ltd. (Shanghai, China). The main materials and chemical reagents used in the experimental process are shown in Table 2.

### 2.2. Preparation

Firstly, the T2 Cu alloy was cleaned and sanded so that the spray suspension could adhere to the alloy better. Secondly, the sanded and polished T2 Cu alloy was put into anhydrous ethanol and deionized water, respectively, for 10 min of ultrasonic cleaning to remove impurities and grease. Thirdly, 10 mL N-hexane organic solvent, 1 g PDMS, 0.1 g silicone rubber curing agent, and different contents of aluminum nanoparticles were mixed to modulate the suspension. Meanwhile, the suspension’s homogeneity was guaranteed using magnetic stirring at 400 r/min for 120 min. Fourthly, the cleaned T2 copper metal was placed in a 100 °C oven and preheated for 10 min in a vacuum-drying oven. Fifthly, the preheated T2 Cu alloy sheet was fixed, and the evenly stirred suspension was transferred to the spray gun. During the process of air spraying, the spraying pressure was set to 0.4 MPa, and the spraying distance was set to 15–20 cm. Finally, the sprayed samples were placed in a constant-temperature oven at 100 °C for 1 h and then cooled to room temperature to obtain the final samples. In the preparation process, the polishing process effectively removes the surface oxide layer of the alloy. Meanwhile, the vacuum treatment also effectively reduces the influence of sample oxidation. In addition, sample defects such as flow marks, bottom leakages, pinholes, and bubbles often occur in the preparation process. To effectively avoid the above-mentioned sample defects, the distance between the spray gun and sample, the spray gun’s movement speed, the cleanliness of the paint, as well as various process parameters must be strictly controlled during the spraying process. At the same time, repeating the spraying tests multiple times also ensures the acquisition of high-quality samples.

### 2.3. Characterization

The elemental composition of the coating surface was analyzed using an energy-dispersive X-ray spectrometer (EDX, Oxford Instrument Inca X-MAX, Singapore). The static contact angle and rolling angle of the coating surface were measured using a contact angle meter (OCA20, Dataphysics GmbH, Filderstadt, Germany). The corrosion resistance of the prepared samples was measured using an electrochemical workstation (CHI660D, CH Instruments Company, Shanghai, China). Electrochemical impedance spectroscopy (EIS) was used to perform tests between 10 mHz and 100 kHz. And the polarization curve was recorded between −600 and 200 mV.

## 3. Results and Discussion

### 3.1. Coating Wettability Test

**Figure 1:**
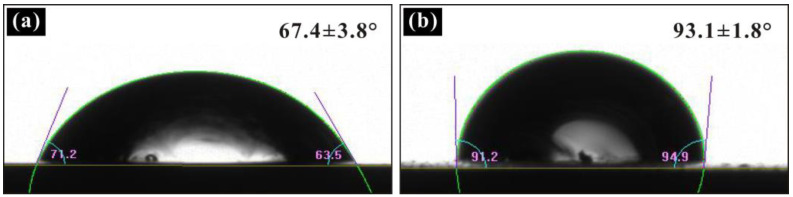
(**a**) The wettability test results for the T2 Cu alloy and (**b**) the wettability test results for the surface of the T2 Cu alloy sprayed with PDMS.

The wettability test results for the T2 Cu alloy show that the bare alloy does not exhibit hydrophobicity, as shown in Figure 1a. Furthermore, the static contact angle of the water droplets on the surface of the T2 Cu alloy is only 67.4 ± 3.8°, as shown by fitting the image of the water droplets, which means that in this state, the solid–liquid contact area ratio reached a high proportion. Figure 1b shows the wettability test results for the surface of the T2 Cu alloy sprayed with PDMS. It can be seen that the static contact angle of the water droplets on the surface increases to 93.1 ± 1.8°. Compared with the bare alloy, the hydrophobicity of the PDMS-coated samples is significantly increased. Meanwhile, the ratio of solid–liquid contact areas in this state is also reduced to a great extent. This is because PDMS is a polymer, which has heat resistance, cold resistance, small variation in viscosity with temperature, waterproofing, a small surface tension, and other advantages [31]. And because of the low surface tension of PDMS, the hydrophobicity of the sample sprayed with this polymer is significantly reduced.

**Figure 2:**
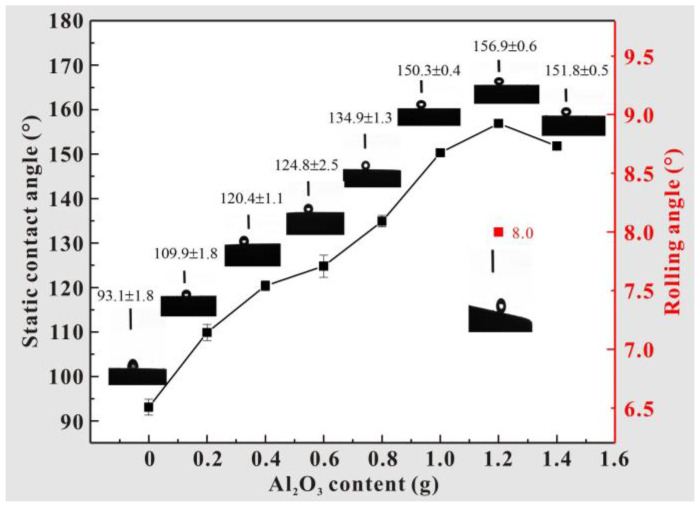
Wettability results for spraying coatings with different aluminum contents.

A multifunctional coating was prepared on the surface of the T2 Cu alloy by air spraying. During the test, the mass of PDMS was set to 1 g, and the amount of silicone rubber curing agent used to solidify the PDMS was 0.1 G. Furthermore, the wettability of the coating surface was optimized by adjusting the content of Al_2_O_3_. Figure 2 shows the result curve of the wetting characteristics of the coating with different contents of Al_2_O_3_. It can be seen that the relationship between the addition of Al_2_O_3_ and the static contact angle is approximately linear in the range of 0–1.2 g. When the content of Al_2_O_3_ reaches 1.2 g, the maximum contact angle is 156.9 ± 0.6°, and the rolling angle is less than 10°, which shows excellent superhydrophobic characteristics. When the content of Al_2_O_3_ further increases to 1.4 g, the contact angle begins to decrease, and the rolling angle disappears. Thus, in subsequent experiments, 1.2 g of Al_2_O_3_ is set as the optimal amount to be added, and the coating in this state is labeled as an Al_2_O_3_-PDMS composite coating in the following sections.

**Figure 3:**
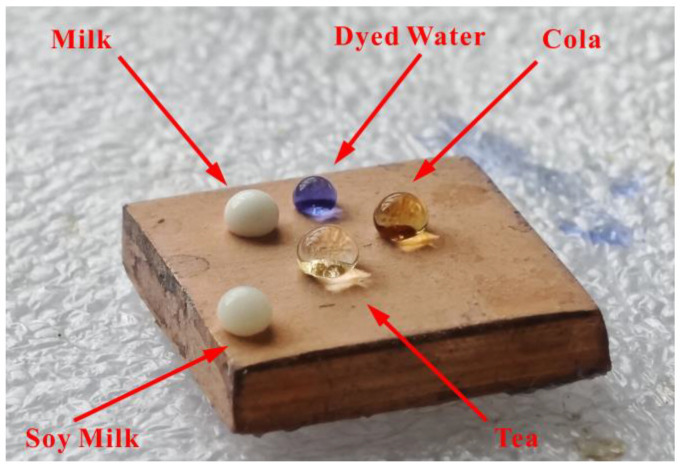
Wettability of the common liquids on the Al_2_O_3_-PDMS composite coating surface.

The prepared Al_2_O_3_-PDMS composite coating still shows good liquid displacement performance for many kinds of common liquid media. As shown in Figure 3, tea, milk, soy milk, and cola all exhibit a nearly spherical droplet-like effect on the surface of the Cu alloy sprayed with an Al_2_O_3_-PDMS composite coating, as with the superhydrophobic effect described above. Furthermore, the contact angle meter was used to quantitatively analyze the wettability of these liquids. And the corresponding measurement results are shown in Table 3, where it is clear that the static contact angles of these liquids are all greater than 150°, and the rolling angles are all less than 10°, which shows good droplet displacement.

**Figure 4:**
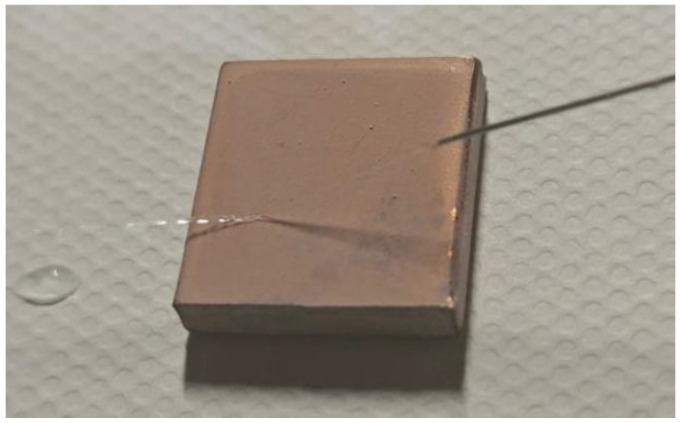
Results of flow reflection test of Al_2_O_3_-PDMS composite coating surface.

In order to further test the wettability of the prepared Al_2_O_3_-PDMS composite coating, a syringe was used to inject water into the surface of the sample. The corresponding test results are shown in Figure 4. It can be seen that the injected water is completely reflected off the surface when it comes into contact with the Al_2_O_3_-PDMS composite coating, and the reflection angle is slightly lower than the incident angle. Meanwhile, no droplet residue was observed during the experiment. This phenomenon indicates that the prepared coating has a very low surface energy and almost no adhesion to the liquid.

### 3.2. Coating Morphology Analysis

**Figure 5:**
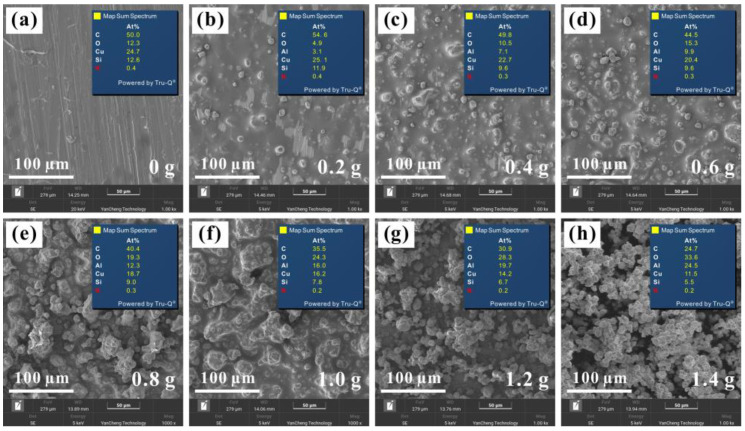
Surface morphologies of Al_2_O_3_-PDMS composite coatings with different aluminum contents: (**a**) 0 g, (**b**) 0.2 g, (**c**) 0.4 g, (**d**) 0.6 g, (**e**) 0.8 g, (**f**) 1.0 g, (**g**) 1.2 g, (**h**) 1.4 g.

**Figure 6:**
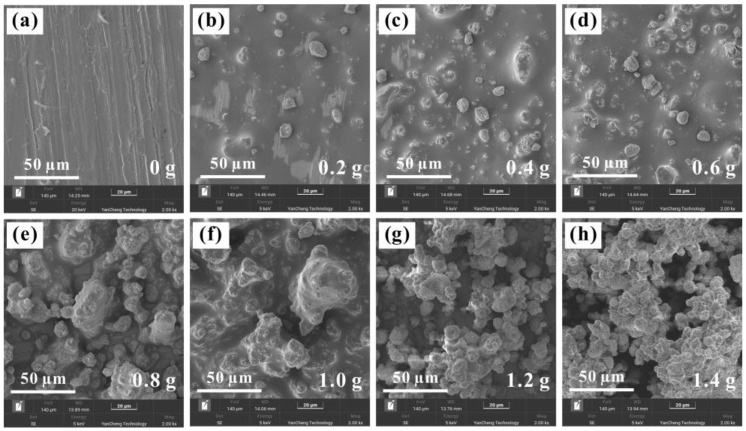
Surface morphologies of amplified Al_2_O_3_-PDMS composite coatings with different aluminum contents: (**a**) 0 g, (**b**) 0.2 g, (**c**) 0.4 g, (**d**) 0.6 g, (**e**) 0.8 g, (**f**) 1.0 g, (**g**) 1.2 g, (**h**) 1.4 g.

**Figure 7:**
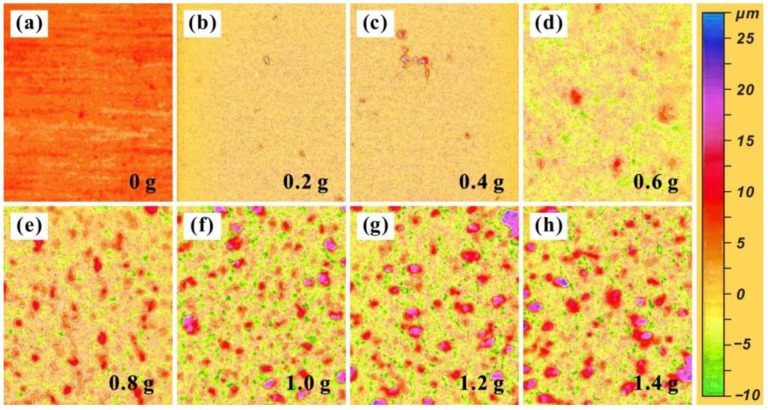
Surface roughness of Al_2_O_3_-PDMS composite coatings with different aluminum contents: (**a**) 0 g, (**b**) 0.2 g, (**c**) 0.4 g, (**d**) 0.6 g, (**e**) 0.8 g, (**f**) 1.0 g, (**g**) 1.2 g, (**h**) 1.4 g.

Through the above experiments, the Al_2_O_3_-PDMS composite coating with excellent hydrophobic function was successfully prepared on the surface of the T2 Cu alloy. Furthermore, Figure 5 and Figure 6 show the surface morphologies of the coatings at different magnifications with different contents of Al_2_O_3_. It can be found that, with the increase in aluminum content, the atomic percentage of the aluminum element gradually rises, confirming the successful preparation of the Al_2_O_3_-PDMS composite coating. Meanwhile, as shown in Figure 7, the surface roughness of Al_2_O_3_-PDMS composite coatings with 0, 0.2, 0.4, 0.6, 0.8, 1.0, 1.2, and 1.4 g Al_2_O_3_ contents is 0.62, 0.87, 1.20, 2.41, 4.38, 5.59, 7.23, and 7.20 μm, respectively. In addition, the relationship between the addition of Al_2_O_3_ and the roughness of the coating surface is approximately linear in the range of 0–1.4 g. The morphologies of the coatings prepared by air spraying all show layered granular micro–nano-structures, which play an important role in achieving superhydrophobicity on the surface of the Cu alloy [32]. When the content of Al_2_O_3_ reaches 1.2 g, the surface is rougher than that of the bare alloy, and thus, the hydrophobic effect is better. However, when the content of Al_2_O_3_ increases to 1.4 g, a denser coating is formed on the surface, which leads to a slight decrease in surface roughness. Therefore, in this state, the hydrophobicity shows a slight downward trend.

**Figure 8:**
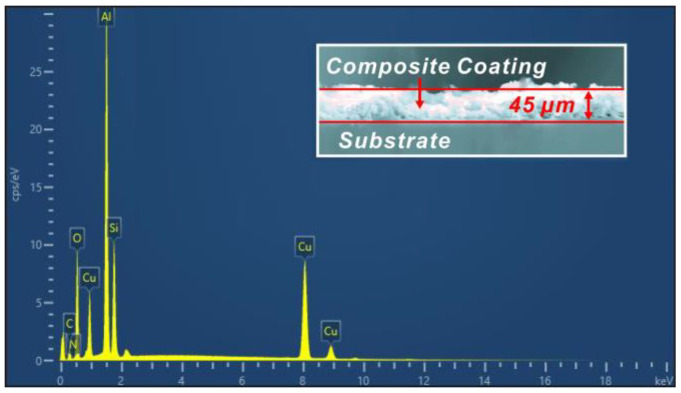
EDS spectrum of Al_2_O_3_-PDMS composite coating with 1.2 g Al_2_O_3_.

After obtaining a micro-structure on the surface of the prepared Al_2_O_3_-PDMS composite coating with 1.2 g Al_2_O_3_ on the T2 Cu alloy, further analysis was conducted on the elemental characteristics of such a superhydrophobic surface. Figure 8 shows the EDS spectrum of the Al_2_O_3_-PDMS composite coating. It can be found that the coating obtained by air spraying is mainly composed of C, N, O, Cu, Al, and Si elements. It can also be inferred that the coating surface is filled with a large amount of Al elements, which stem from the addition of Al_2_O_3_ ceramic particles. Meanwhile, PDMS with a thickness of about 45 μm covers the T2 Cu alloy’s surface and serves as an important bone support for the physical structure of the Al_2_O_3_-PDMS composite coating.

**Figure 9:**
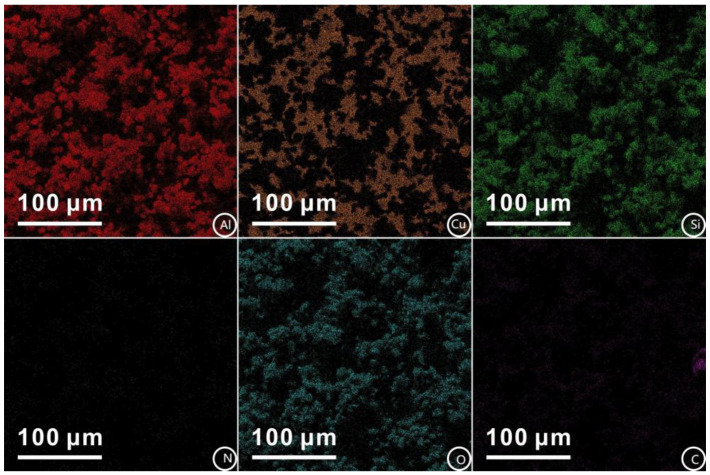
Mapping scanning results of Al_2_O_3_-PDMS composite coating.

The mapping scanning results of the main elements of the Al_2_O_3_-PDMS composite coating are shown in Figure 9. In can be seen that the elements are evenly distributed. Meanwhile, each element exists in a certain proportion and presents different structural characteristics. It is because of this layered structure that the superhydrophobic characteristic of the coating can be achieved. This is because the micro–nano-structure coating prepared by air spraying has changed the state of the highly viscous solid–liquid contact interface. Instead, the interface characteristic is the a solid–gas–liquid three-phase composite contact state (Cassie Model) [33]. Under this condition, the prepared micro–nano-structure can capture more air phases and then produce an “air cushion” effect to hold up the water droplets. Ultimately, the viscous resistance to water nearly disappears, thus showing excellent superhydrophobic characteristics. This is due to the fact that the viscous resistance refers to the resistance generated by fluid viscosity when an object moves in it. Viscous resistance affects superhydrophobic behavior by influencing the interaction between the droplet and solid surface. Research has reported that when a droplet collides with the superhydrophobic surface, the lower the viscous resistance is, the faster the liquid film tends to spread and retract [34]. At the same time, the droplet will also rebound earlier at a higher height. Therefore, it exhibits a better repellent effect on the liquid droplet, which means a better superhydrophobic effect.

**Figure 10:**
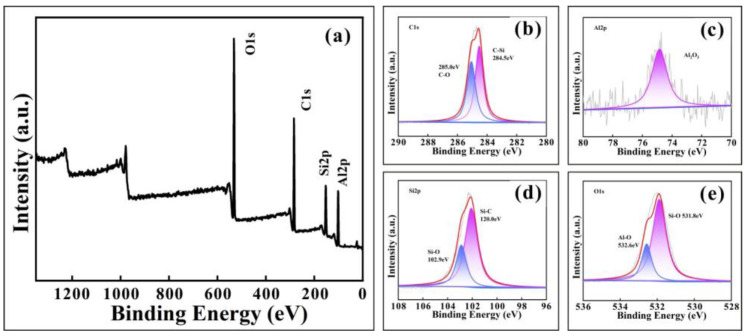
Full XPS spectrum and each part of the fitting atlas of the Al_2_O_3_-PDMS composite coating: (**a**) the full XPS spectrum, (**b**) C1s, (**c**) Al2p, (**d**) Si2p, (**e**) O1s.

In addition, the full XPS spectrum and each part of the fitting atlas of the Al_2_O_3_-PDMS composite coating are shown in Figure 10. The four main characteristic peaks of Al2p (102.08 eV), Si2p (153.08 eV), C1s (284.08 eV), and O1s (532.08 eV) can be seen clearly in the full XPS spectrum. The Al2p’s single-peak binding energy of Al_2_O_3_ is 74.88 eV, which is close to the binding energy of Al_3_O_2_ in the literature. The C-O single bond and C-Si single bond are obtained by fitting the peaks of C1s, and the peak values are 285.0 eV and 284.5 eV, respectively. The fitting peak values of O1s are 531.8 eV and 532.6 eV, which correspond to the Si-O bonds in PDMS and Al-O bonds in Al_2_O_3_, respectively. The XPS results confirm that the formation of an Al_2_O_3_-PDMS composite coating with superhydrophobic behavior has been successfully achieved.

### 3.3. Multifunctional Behavior Testing

**Figure 11:**
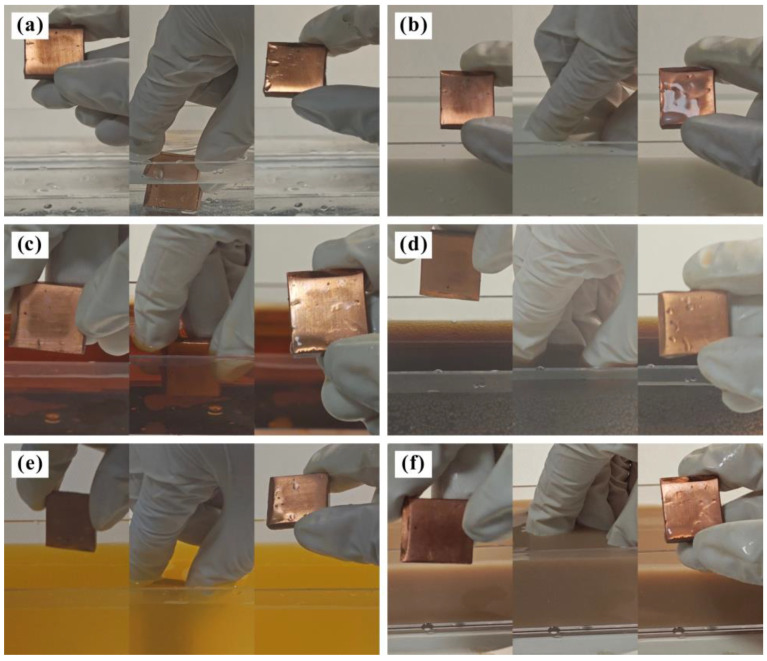
Anti-pollution experiment with T2 Cu alloy against different pollution media: (**a**) water, (**b**) milk, (**c**) tea, (**d**) cola, (**e**) juice, and (**f**) mud.

In daily life, the surface energy of common metals is high, which means that the liquid is always in a hydrophilic spreading state when it comes into contact with the metal [35]. In this experiment, water, milk, tea, cola, juice, and mud were used as pollution sources to test the anti-pollution behavior of the Al_2_O_3_-PDMS composite coating. Specifically, the sample was submerged in the liquid in a vertical fashion and held for 3 s before being removed. The test was repeated three times, and the surface state of the sample was recorded. Figure 11 shows the anti-pollution experiment with the T2 Cu alloy against different pollution media. It can be seen that after the samples are taken out of the pollution media, more solutions are adhered to the surface. This phenomenon indicates that the bare alloy without any treatment cannot exhibit any anti-pollution functions, which is due to its hydrophilic characteristic.

**Figure 12:**
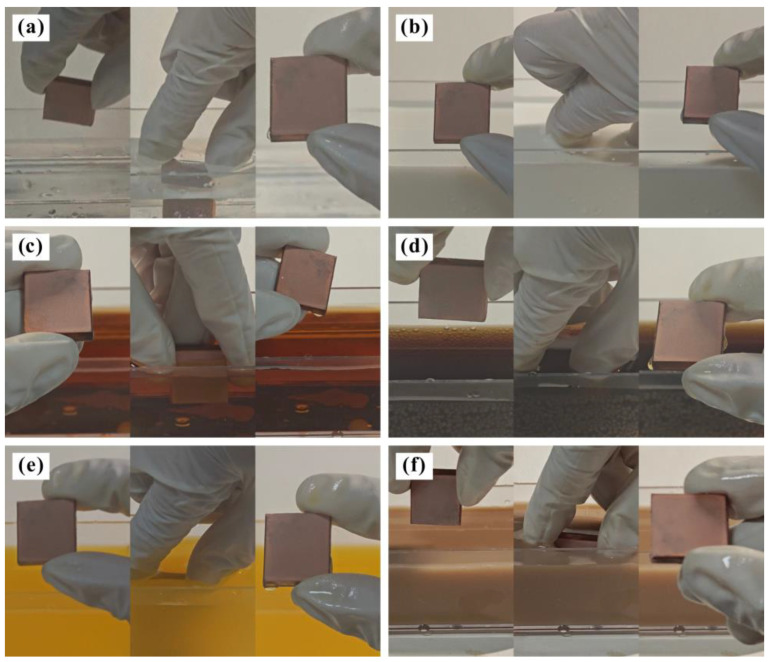
Anti-pollution experiment with Al_2_O_3_-PDMS composite coating against different pollution media: (**a**) water, (**b**) milk, (**c**) tea, (**d**) cola, (**e**) juice, and (**f**) mud.

Figure 12 shows the anti-pollution experiment with the Al_2_O_3_-PDMS composite coating against water, milk, tea, cola, fruit juice, and mud. It can be seen that after the samples are taken out of the pollution media, there is no solution adhering to the surface. This phenomenon indicates that the T2 Cu alloy treated by air spraying exhibits good anti-pollution function, which is due to the superhydrophobic characteristic of the coating. The interface property of the micro–nano-structure coating with a liquid is a solid–gas–liquid three-phase composite contact state (Cassie Model). This state can capture more air phases and then produce an “air cushion” effect to hold up the pollution media [36]. Ultimately, the viscous resistance to pollution media is almost eliminated, thus exhibiting excellent anti-pollution behavior.

**Figure 13:**
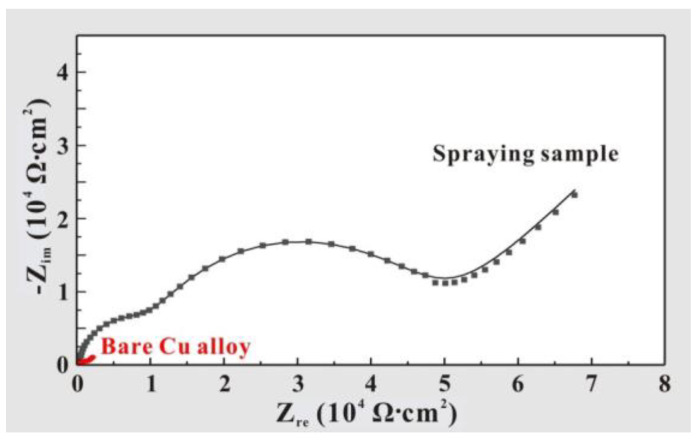
Electrochemical impedance spectroscopies of T2 Cu alloy and Al_2_O_3_-PDMS composite coating.

In order to test the corrosion resistance of the prepared samples, the electrochemical impedance spectroscopy results for the T2 Cu alloy and Al_2_O_3_-PDMS composite coating are shown in Figure 13. It can be seen that the arc radius of the capacitive resistance curve of the Al_2_O_3_-PDMS composite coating is much larger than that of the bare alloy. It is well known that in the impedance spectrum, the larger the diameter of the capacitive arc is, the larger the charge transfer resistance (Rct) of the sample is [37]. And the larger the Rct value is, the stronger the corrosion resistance of the sample is [38]. As can be seen from Figure 13, the Rct value of the bare alloy is about 2000 Ω⋅cm2, while the Rct value of the Al_2_O_3_-PDMS composite coating is as high as 12,000 Ω⋅cm2. Therefore, from the perspective of charge transfer resistance, the corrosion resistance of the T2 Cu alloy has been significantly improved after spraying with a coating. In addition, the comparisons of impedances with other reported coatings obtained through spraying processes are also demonstrated in Table 4 [39,40,41], suggesting that the Al_2_O_3_-PDMS composite coating prepared in this work offers improved corrosion resistance.

**Figure 14:**
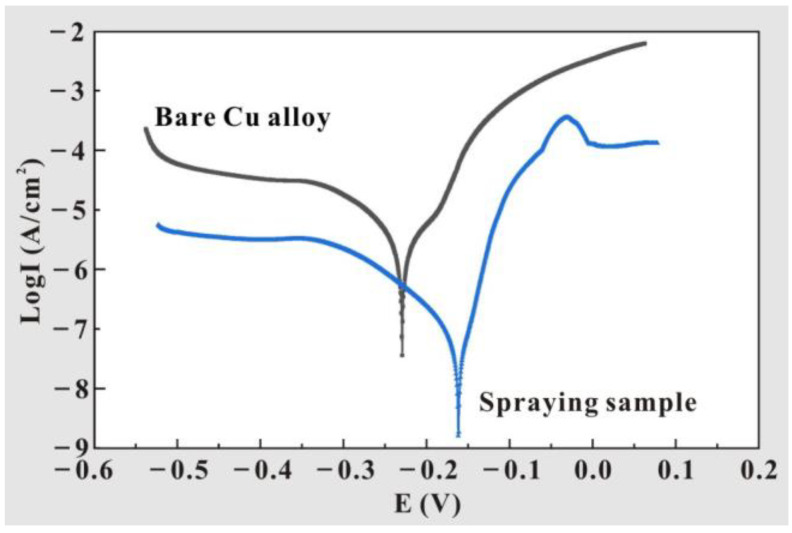
Potentiodynamic polarization curves of T2 Cu alloy and Al_2_O_3_-PDMS composite coating.

For the purpose of further verifying the corrosion resistance of the prepared samples, the potentiodynamic polarization curves of the T2 Cu alloy and Al_2_O_3_-PDMS composite coating were tested, as shown in Figure 14. The corresponding electrochemical parameters are also presented in Table 5. It is well known that in the polarization curve, the smaller the corrosion current density is and the higher the corrosion potential is, the stronger the corrosion resistance of the sample is [42]. It can be seen in Table 5 that compared with the bare alloy, the corrosion current density of the Al_2_O_3_-PDMS composite coating decreases from 3.61 × 10^−8^ to 1.60 × 10^−9^ A/cm^2^, and the corrosion potential increases from −0.23 to −0.16 V. This means that after the coating is sprayed on the T2 Cu alloy, its corrosion resistance has been significantly improved. Meanwhile, the corrosion resistance improvement rate (IE) of the coating sample can be calculated using the following formula [43]:

IE = (𝐼_bare_−𝐼_coating_)𝐼_bare_∗100% (1)

According to this formula, it can be calculated that the corrosion resistance improvement rate of the coating sample reaches 95.6% compared to the bare sample. The significant improvement in corrosion resistance is due to the combined effect of two factors. Firstly, the Al_2_O_3_-PDMS composite coating has a good physical isolation effect on the corrosive medium. Secondly, the prepared micro–nano-structure enhances the entrapment of air phases, which strengthens the expulsion effect on corrosive media, further enhancing the corrosion resistance.

**Table 5:** Electrochemical parameters for T2 Cu alloy and Al_2_O_3_-PDMS composite coating.

Samples	Corrosion Potential (V)	Corrosion Current Density (A/cm^2^)
T2 Cu alloy	−0.23	3.61 × 10^−8^
Al_2_O_3_-PDMS composite coating	−0.16	1.60 × 10^−9^

**Figure 15:**
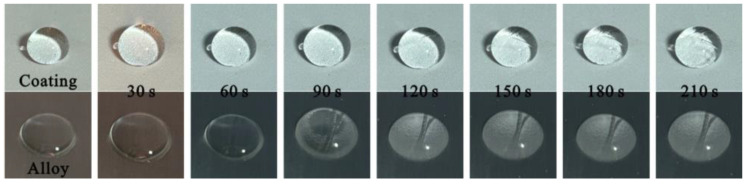
Freezing process of a 20 μL water droplet on T2 Cu alloy and Al_2_O_3_-PDMS composite coating.

In addition, to test the anti-icing performance of the prepared samples, Figure 15 records the freezing process of a 20 μL water droplet on the T2 Cu alloy and Al_2_O_3_-PDMS composite coating. It can be seen that the water droplet on the bare alloy completely solidifies at 150 s. However, the water droplet on the coating does not fully solidify until 210 s, indicating that the coating prepared on the Cu alloy has excellent ice delay performance. This is because the prepared micro–nano-structure can capture a large amount of air, which makes the heat transfer coefficient of the coating surface much lower than that of the Cu alloy substrate, thus achieving a delaying effect in the icing process. The above research indicates that a superhydrophobic, corrosion-resistant, and anti-icing coating was successfully prepared on a Cu alloy’s surface using one-step air spraying technology. Such air spraying is a commonly used coating method, which is a technology that uses compressed air to atomize paint for spraying. Its advantage lies in the ability to freely select spraying conditions, such as the paint quantity, paint bundle shape, paint bundle diameter, paint particle size, air pressure, and other parameters. Meanwhile, the process is relatively easy to carry out, and the cost is relatively low. It is also applicable to the field of large-scale preparation. As a technology, it also has certain drawbacks, such as a low paint utilization rate and easy carrying of water and oil in air, which can affect the quality of the coating formation.

## 4. Conclusions

(1) Using air spraying technology, a micro–nano-structured coating with layered characteristics was prepared on the surface of a T2 Cu alloy. By adjusting the content of Al_2_O_3_, the wettability of the coating surface was optimized. When the content of Al_2_O_3_ reaches 1.2 g, the contact angle obtains a maximum value of 156.9 ± 0.6°, and the rolling angle is less than 10°, thus exhibiting excellent superhydrophobic characteristics.
(2) The structural coating prepared by air spraying changed the state of the high-viscosity solid–liquid contact interface. Instead, the interface characteristic is a composite three-phase contact state of solid–gas–liquid. Under this condition, the prepared micro–nano-structure can capture more air phases and then produce an “air cushion” effect to hold up the liquid. Ultimately, the viscous resistance of the coating to water nearly disappears, thus exhibiting excellent superhydrophobic characteristics, anti-pollution behavior, and corrosion resistance.
(3) The water droplet on the Cu alloy substrate completely solidifies and freezes at 150 s. However, the water droplet on the Al_2_O_3_-PDMS composite coating does not fully freeze until 210 s. This is because the prepared micro–nano-structure can capture a large amount of air, which makes the heat transfer coefficient of the coating surface much lower than that of the Cu alloy substrate, thus achieving a delay effect in the icing process.
(4) For future work, it is necessary to gain a deeper understanding of the anti-icing mechanism of as-prepared superhydrophobic coatings by using numerical simulation. A new simulation model should also be developed to predict the anti-icing performance of as-prepared coatings under different conditions, thus providing valuable theoretical guidance for experimental research. Meanwhile, the fractional description of thermal transport could be useful if such as-prepared nanoscale samples are considered for future research [44].

## Figures and Tables

**Table 1:** The corresponding chemical compositions of the T2 Cu alloy plate.

Element	Cu	Ag	O	Sb	As	Fe	Pb	S	Others
Mass fraction (wt%)	99.90	0.001	0.06	0.002	0.002	0.005	0.005	0.005	0.02

**Table 2:** Main materials and chemical reagents used in the experimental process.

Materials	Specifications
Aluminum oxide particles	50 nm, 99.8%
Polydimethylsiloxane	SYLGARD™ 184
Silicone rubber curing agent	SYLGARD™ 184
N-hexane	97.0%

**Table 3:** Static contact angles and rolling angles of common liquids on Al_2_O_3_-PDMS composite coating surface.

Liquid	Static Contact Angle (°)	Rolling Angle (°)
Water	156.3 ± 0.6	8.0 ± 0.2
Tea	156.4 ± 0.3	8.5 ± 1.0
Milk	151.2 ± 2.0	8.9 ± 1.1
Soy milk	154.4 ± 1.4	8.5 ± 0.8
Cola	155.7 ± 0.4	8.1 ± 0.5

**Table 4:** Comparisons of corrosion resistances with other reported coatings.

Samples	Electrolyte	R_ct_ (Ω⋅cm ^2^)	Reference
Graphene coating	3.5 wt% NaCl solution	3768	[39]
Cu-AlN coating	3.5 wt% NaCl solution	3200	[40]
Bio-based coating	3.5 wt% NaCl solution	10,670	[41]
Al_2_O_3_-PDMS coating	3.5 wt% NaCl solution	12,000	This work

## Data Availability

The original contributions presented in the study are included in the article, further inquiries can be directed to the corresponding author.
